# Management of Orofacial Infantile Haemangioma: A Case Report

**DOI:** 10.1155/crid/4988221

**Published:** 2024-12-18

**Authors:** Othman Zuhir, Nurhidayah Muhd Noor, Aminah Marsom

**Affiliations:** Department of Paediatric Dentistry, Selayang Hospital (Ministry of Health), Batu Caves, Selangor, Malaysia

**Keywords:** benign, case report, dentistry, haemangioma, infancy, neoplasm, tumour, vascular

## Abstract

Infantile haemangioma (IH) is the most common childhood tumour, often developing in the head and neck region. It may cause disfigurement, functional impairment, or tooth developmental issues when it is present in the oral cavity. We report a case of a 2-month-old boy referred to the paediatric dentistry team with a segmental IH involving the left periorbital, cheek, and hard palate. The patient had a maculopapular rash in the periorbital area, a telangiectatic patch on his left cheek, and papules on the left posterior hard palate. He was comanaged with the paediatric, ophthalmology, dermatology, and cardiology teams. He was treated with oral propranolol, resulting in the lesions reducing in size. Early intervention is crucial to prevent severe complications affecting the patient's appearance, function, speech, feeding, and dentition. Dentists should be familiar with the clinical presentations and preventative measures, as we may encounter such patients with oral cavity involvement.

## 1. Introduction

Infantile haemangioma (IH) is the most common benign vascular tumour of infancy, with an incidence between 4% and 5% of infants [1]. Three out of five IH cases affect the head and neck region, with almost two-thirds of these cases involving the orbit, nose, and mouth [2]. While it is often visibly absent at birth, 30% of IH cases exhibit a precursor lesion that presents as a faint telangiectatic cluster, pale vasoconstrictive area, or bluish-grey macule [3].

IH typically becomes clinically apparent within the first few weeks after birth as it undergoes rapid growth during the proliferation phase before entering a spontaneous involution phase over several years [3]. While intervention is unnecessary in most IH cases, 10% of IH cases require treatment as the lesions may cause debilitating complications such as ulceration, disfigurement, and functional impairment [4, 5]. Currently, patients with IH are treated with oral propranolol, a nonselective adrenergic beta-blocker, as the first line of treatment [6]. The management of IH is typically multidisciplinary, encompassing the expertise of various healthcare professionals, including paediatric dentists.

This case report presents a patient with a segmental IH involving the left periorbital, cheek, and hard palate who was managed at our hospital. The patient's presentation highlights the importance of early identification and timely intervention in cases of IH, especially those affecting the oral and maxillofacial region.

## 2. Case Presentation

A 2-month-old male infant was referred to our paediatric dentistry department with a known diagnosis of IH on his left cheek. The mother reported red streakiness on the left cheek on the baby's first day of life. The lesions progressively developed into erythematous crusty macules and swelling over the left cheek within 2 and 3 weeks, respectively. Before this initial appointment, the mother reported that the lesions had rapidly grown towards the left periorbital area.

The patient was born preterm at 36 weeks via caesarean section and weighed 3.18 kg at birth. His mother had gestational diabetes mellitus and hypertension during pregnancy.

### 2.1. Findings

**Figure 1:**
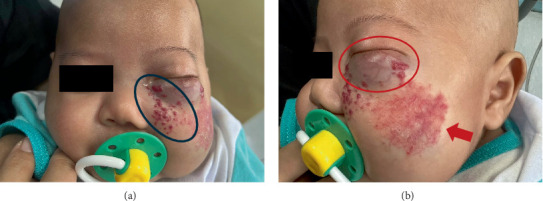
(a) The front view showed the presence of an erythematous maculopapular rash on the left medial cheek (blue circle). (b) The side view demonstrated a diffused swelling in the left lower eyelid with maculopapular lesions (red circle). A telangiectatic patch could also be seen in the left lateral cheek (red arrow).

On examination, the patient exhibited diffused telangiectatic swelling with maculopapular lesions in the left lower eyelid (Figure 1). He had left eye proptosis with congestion of the lower palpebral conjunctiva and multiple smooth lobulated lesions. The visual axis was clear, with no sign of ptosis or amblyopia. There were multiple erythematous maculopapular rashes on the left medial cheek. On the left lateral cheek, a telangiectatic patch with an underlying firm mass measuring 4 by 5 cm was noted. None of the lesions were ulcerated or bleeding. The left nasal dorsum appeared distorted, but there was no nasal obstruction or septal destruction. Intraorally, red papules were palpable on the left posterior hard palate with no pharyngeal extension. An erythematous macule measuring 1 cm by 0.5 cm was observed in the left posterior buccal mucosa. The alveolar ridges appeared normal.

### 2.2. Diagnostic Assessment

**Figure 2:**
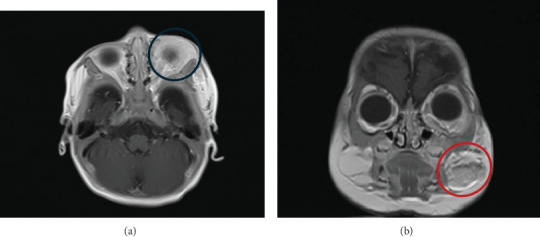
(a) Axial view shows a homogenous left periorbital mass (blue circle), which causes left eye proptosis. (b) The coronal view shows a mass (red circle) at the deep subcutaneous layer of the left cheek region.

A magnetic resonance imaging (MRI) of the orbit revealed a homogenous mass in the left periorbital region involving the left preseptal area with intraorbital extraconal extension to the left infratemporal fossa. Smaller, similar-appearing soft tissue masses were identified in the subcutaneous layer of the left cheek and the deep subcutaneous layer of the left mandibular region (Figure 2). The patient also underwent an abdominal ultrasound, an electrocardiogram (ECG), and echocardiography, which yielded unremarkable findings. Based on the history, examination, and investigations, a diagnosis of the left periorbital, cheek, and hard palate IH of the segmental subtype was confirmed.

### 2.3. Therapeutic Interventions

The patient was managed under an interdisciplinary approach. The paediatric and paediatric dermatology teams assessed the facial skin lesions and any developing lesions in the body and the patient's general condition. The ophthalmology team evaluated the eye for improvement in his proptosis and signs of amblyopia, ptosis, or strabismus. The cardiology team examined the heart condition for any anomalies before starting any treatment.

Upon presentation to our clinic, he was already taking oral propranolol 1.5 mg twice daily for a few days as prescribed by the paediatric team, equivalent to 2 mg/kg/day. As paediatric dentists, we examined the patient's face and oral cavity for signs of ulceration, disfigurement, or functional impairment, such as feeding difficulties and speech issues. We demonstrated a technique of oral stimulation around the oral mucosa using the index finger covered in a wet washcloth. Oral stimulation was deemed essential, even in the absence of teeth, to introduce the patient to an oral hygiene routine. The parents were advised to brush their child's teeth using a baby toothbrush with a tiny smear of fluoride toothpaste once the teeth erupted.

### 2.4. Follow-Up and Outcomes

**Figure 3:**
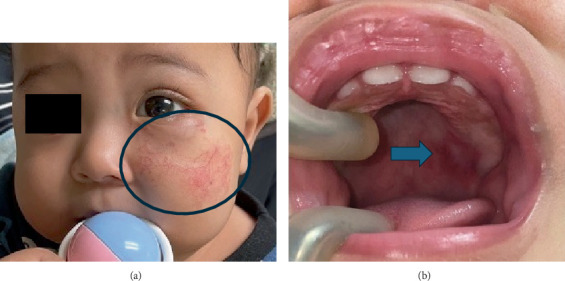
(a) The 9-month-old patient could be seen with faint telangiectasia and maculopapular rash on the left cheek (blue circle). (b) The resolving lesion was visible in the left posterior hard palate (blue arrow).

After the initial appointment, the paediatric team gradually increased the oral propranolol dosage by 0.5 mg/kg/day at each visit, up to a maximum of 3 mg/kg/day given in divided doses. During the first follow-up review in our department after 7 months, the 9-month-old patient appeared cheerful and vocalised during interactions. The swelling on the left lower eyelid, maculopapular rash on the left medial cheek, and telangiectatic patch on the left lateral cheek had decreased in size and colour intensity. The distorted left nasal dorsum had slightly improved, but the patient still had an asymmetrical lip while smiling. Intraorally, the red papules on the left posterior hard palate had decreased in size (Figure 3). No changes were observed in the erythematous macule on the left posterior buccal mucosa. The patient's deciduous central and lateral incisors erupted without any signs of caries.

The mother reported assisting the patient with brushing his teeth once daily using a silicone brush after his teeth began to erupt. However, she found it challenging due to the patient's resistance and uncooperativeness. We then demonstrated a toothbrushing technique, involving both parents sitting knee-to-knee with the patient's head on one parent's lap while the other held and distracted him. If only one parent was available, the patient could sit on the parent's lap while facing forward, with one hand holding the patient and the other brushing their teeth. We emphasised the importance of brushing twice daily. As the patient transitioned to a soft diet and bottled formulated milk, we advised the parents to monitor the sugar content in their child's diet and reduce the snacking frequency between meals. We showed how to wipe the patient's teeth using a wet washcloth, especially after eating or taking medication. The parents were also advised to avoid feeding the patient to sleep with a milk bottle, as prolonged sugar exposure can contribute to early childhood caries.

**Figure 4:**
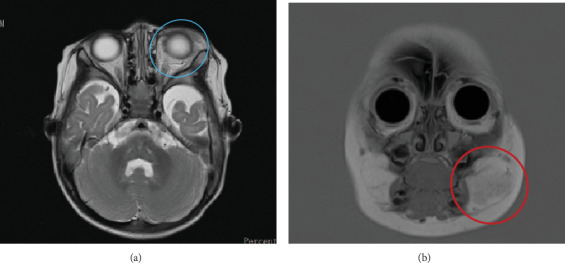
(a) Axial view of the brain MRI taken 7 months after the patient started oral propranolol, indicating a smaller left periorbital mass (blue circle). (b) The coronal view of the brain MRI showed a less defined subcutaneous mass in the left cheek region (red circle).

Before the first follow-up visit, a brain MRI was taken (Figure 4) and showed that the masses on the left periorbital and left cheek were smaller than previously seen, suggesting the involution phase. Additionally, a head and neck magnetic resonance angiography (MRA) was performed, which confirmed the absence of posterior fossa malformation or arterial anomalies. These findings and the lack of cardiac and eye anomalies ruled out the diagnosis of PHACE (posterior fossa malformations, haemangioma, arterial anomalies, coarctation of the aorta/cardiac defects, and eye abnormalities) syndrome, which is characterised by segmental haemangiomas with various developmental defects, including posterior fossa anomalies, arterial anomalies, cardiac anomalies, and eye anomalies.

**Figure 5:**
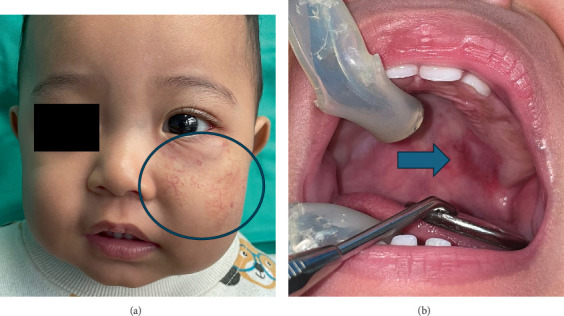
(a) The 11-month-old patient had lesions on the left cheek (blue circle). (b) The erythematous macule on the left posterior buccal mucosa was similar to the previous visit (blue arrow).

During the second follow-up visit 3 months later, the swelling in the left lower eyelid had further reduced (Figure 5). Several faint red macules with flat telangiectatic patches were present in the left medial and lateral cheek, demonstrating signs of improvement. The distortion of the left nasal dorsum had improved. Intraorally, no changes were seen in the erythematous patch on the hard palate and the macule on the left buccal mucosa. His deciduous dentition remained sound. The mother reported assisting with toothbrushing twice daily using a baby toothbrush and a smear of fluoride toothpaste as advised. The patient had transitioned to a semisolid diet and drank 5 oz of formulated milk six times daily from a bottle. As the patient turned one, we encouraged the parents to introduce a sippy cup to wean the patient off bottle-feeding. However, they were advised to fill the cup with only water, unless during mealtimes. The patient had also started to vocalise words like “mama” and “abah.” We encouraged the parents to monitor their child's speech development and report any speech impediment. Finally, we applied fluoride varnish to his teeth to protect against caries and scheduled a follow-up appointment in 6 months.

During a separate appointment on the same day, the paediatric team also met with the parents to address their concern about the child's appearance if scarring and facial asymmetry persisted. The parents were informed that while further treatment was not currently recommended, there was an option to consider laser treatment to remove scarring from the child's face once he grew older if the lesion did not completely disappear.

## 3. Discussion

This case report describes a patient with a segmental IH affecting the left periorbital area, cheek, and hard palate. The patient was successfully treated using a multidisciplinary approach, which included oral propranolol treatment and support from other healthcare professionals. Although paediatricians and dermatologists typically take the lead, dentists, especially paediatric dentists, provide unique expertise in assessing oral complications, offering specific oral hygiene guidance, and monitoring for potential long-term effects on facial and dental development.

The prevalence of IH is influenced by several risk factors such as preterm delivery, low birth weight, preeclampsia, and older maternal age [1]. Approximately one in four preterm infants weighing less than 1 kg is affected by IH [1]. Our patient was born prematurely with a healthy birth weight, but the mother had a history of gestational hypertension. These conditions may cause tissue hypoxia during foetal and infant development, leading to the induction and proliferation of endothelial progenitor cells (EPCs) mediated by the factor HIF-1*α* (hypoxia-inducible factor 1-alpha) [5]. Although prenatal diagnosis may not be possible due to the lesion only appearing and proliferating after birth, postnatal evaluation can provide valuable information to guide management and treatment.

IH growth may manifest clinically as a focal, multifocal, segmental, or indeterminate lesion based on its morphology and distribution [2]. These vascular tumours are frequently found in perioral and intraoral regions such as the lip, cheek, tongue, buccal mucosa, palate, and frenum [6]. In this case, the extent and nature of the lesion were confirmed by clinical and radiographical examinations, and a biopsy was deemed unnecessary for diagnosing IH. While most IH lesions undergo spontaneous regression after several years [3], a facial tumour with segmental morphology, similar to the one affecting our patient, has high treatment needs due to its potential complications [1]. One such complication is a higher propensity to develop ulceration compared to other subtypes [1]. Although the patient did not exhibit any ulceration in this case, a thorough oral cavity examination by a paediatric dentist is essential to identify and manage any possible ulceration. After treatment with an oral propranolol dosage of up to 3 mg/kg/day, the patient demonstrated remarkable improvement. Despite lesion regression, some degree of facial asymmetry remained in the nose and cheek. As such, parents should be informed that distortion of facial structures such as the eye, nose, or oral cavity may persist and be permanent even after adequate treatment and involution [1]. For persistent telangiectasias, laser treatment can be considered after the child has grown older, around the age of 8 or 9 years old [5]. Meanwhile, the development of facial features should be monitored for further improvement.

In addition to the risks of ulceration and disfigurement, perioral and intraoral IH lesions can also impact the patient's speech [5, 7]. Although our patient has not yet started speaking, the parents have been advised to watch for any signs of speech developmental delay or impediments. As paediatric dentists, it is crucial for us to identify speech issues and refer patients to speech therapy services for appropriate support. Early intervention is essential to address any speech challenges and ensure optimal communication development.

IH lesions can also affect the patient's ability to feed due to discomfort, resulting in dysphagia [7]. The difficulty in swallowing can result in food stagnation in the floor of the mouth and the lower teeth [8]. The food stagnation may be worsened by the presence of intraoral lesions, as oral hygiene care can be uncomfortable. This issue may persist even after lesion involution, as fibrous scar tissue in the area may still complicate the oral hygiene routine [8]. Consequently, such patients are susceptible to developing early childhood caries. Moreover, the propranolol syrup introduced to these young patients often contains a sucrose-based excipient that can exacerbate the problem [9]. As an adrenergic beta-blocker, propranolol may also contribute to dental caries by causing decreased salivary flow. Hence, it is crucial for parents to be aware of the risk of early childhood caries and to implement preventive measures, such as cleaning their child's teeth after eating and taking medication; avoiding adding sugar to their child's food, milk, and drinks [10]; and encouraging the use of free-flow cups from 5 months of age.

Several reports have described the findings of enamel hypoplasia in patients with intraoral IH lesions [8, 11]. The hypoxic condition that gave rise to IH can interfere with tooth development and cause enamel hypoplasia [12]. Salanitri and Seow have reported that premature babies with low birth weight have a higher prevalence of enamel hypoplasia. Enamel hypoplasia increases dental sensitivity and predisposes teeth to caries and tooth wear [12]. As such, dentists should focus on early detection and preventive care. Fluoride varnishes or other remineralising agents, such as casein phosphopeptide amorphous calcium phosphate (CPP-ACP), can be applied three or six monthly to assist remineralisation of hypomineralised surfaces and early carious lesions [12]. Parents should also brush the patient's teeth twice daily with a smear of toothpaste containing at least 1000 ppm fluoride [10]. By implementing these strategies, we can help mitigate the oral health challenges associated with IH lesions and ensure optimal dental outcomes for affected patients.

Beyond these points, it is important to consider the potential impact of IH on the patient's psychosocial well-being. Facial disfigurement and functional limitations can significantly affect a child's self-esteem, body image, and social interactions. These psychological challenges can lead to feelings of isolation, anxiety, depression, and low self-worth. Providing emotional support and counselling to both the patient and their family is essential to helping them deal with these emotional challenges and develop positive coping strategies. Addressing the psychological aspects of IH can greatly improve the overall quality of life for affected children and their families.

## 4. Conclusion

This case report demonstrates the pivotal role of dentists in the successful management of IH, particularly when the lesions involve the oral and maxillofacial regions. The patient in this case experienced significant improvement in facial appearance and function following treatment with oral propranolol within a multidisciplinary approach. Early intervention is crucial to prevent complications that can affect a patient's appearance, function, speech, feeding, and dentition. Dentists have unique expertise in assessing oral complications, providing targeted oral hygiene instruction, and monitoring for potential long-term effects on facial appearance and dental development. Therefore, as dentists, it is important that we become familiar with the clinical presentations and preventative measures, as we may encounter such patients with oral cavity involvement. By providing timely and comprehensive care, we can optimise outcomes for patients with IH and improve their overall quality of life.

## 5. Parents' Perspective

We have never heard of this condition previously. After our son was born, he developed red spots on his face. We were concerned upon seeing them. We thought they were due to milk deficiency and that they would resolve themselves. When the lesions started to grow, we were worried that they would affect his internal organs. So, we went to a private clinic to ask about it. They mentioned “strawberry naevus.” I did not know about it, as there was no family history. So, we went to Google to find out more. We were slightly sad when we realised that he had to undergo many investigations, especially when we knew he had to be put to sleep for the MRI.

We felt relieved to find that there was a medication. We had no qualms about our son taking propranolol so long as the swelling reduced. Initially, there was some doubt as we learnt that propranolol was meant to treat hypertension. So far, there was no issue even after the dose was increased. He had no problem taking the medication using the syringe provided. So far, we are happy with all treatments and seeing the improvements. Our message to other parents is to be patient with God's trials. Do not give up because the team takes great care to cure this condition. We want to thank everyone for trying their best to help us as we can see the positive change. The doctors explained to us regarding this condition, so we felt reassured. We appreciate all the hard work put in by the team.

## Data Availability

Information and data used in this case report are available from the corresponding author on reasonable request. They are not publicly available due to ethical issues.
